# The Cholecystectomy As A Day Case (CAAD) Score: A Validated Score of Preoperative Predictors of Successful Day-Case Cholecystectomy Using the CholeS Data Set

**DOI:** 10.1007/s00268-019-04981-5

**Published:** 2019-04-23

**Authors:** A. M. El-Sharkawy, N. Tewari, R. S. Vohra, CholeS Study Group, West Midlands Research Collaborative, R. S. Vohra, S. Pasquali, A. J. Kirkham, P. Marriott, M. Johnstone, P. Spreadborough, D. Alderson, E. A. Griffiths, S. Fenwick, M. Elmasry, Q. Nunes, D. Kennedy, R. Basit Khan, M. A. Khan, C. J. Magee, S. M. Jones, D. Mason, C. P. Parappally, P. Mathur, M. Saunders, S. Jamel, S. Ul Haque, S. Zafar, M. H. Shiwani, N. Samuel, F. Dar, A. Jackson, B. Lovett, S. Dindyal, H. Winter, T. Fletcher, S. Rahman, K. Wheatley, T. Nieto, S. Ayaani, H. Youssef, R. S. Nijjar, H. Watkin, D. Naumann, S. Emeshi, P. B. Sarmah, K. Lee, N. Joji, J. Heath, R. L. Teasdale, C. Weerasinghe, P. J. Needham, H. Welbourn, L. Forster, D. Finch, J. M. Blazeby, W. Robb, A. G. McNair, A. Hrycaiczuk, A. Charalabopoulos, S. Kadirkamanathan, C. B. Tang, N. V. Jayanthi, N. Noor, B. Dobbins, A. J. Cockbain, A. Nilsen-Nunn, J. de Siqueira, M. Pellen, J. B. Cowley, W. M. Ho, V. Miu, T. J. White, K. A. Hodgkins, A. Kinghorn, M. G. Tutton, Y. A. Al-Abed, D. Menzies, A. Ahmad, J. Reed, S. Khan, D. Monk, L. J. Vitone, G. Murtaza, A. Joel, S. Brennan, D. Shier, C. Zhang, T. Yoganathan, S. J. Robinson, I. J. McCallum, M. J. Jones, M. Elsayed, L. Tuck, J. Wayman, K. Carney, S. Aroori, K. B. Hosie, A. Kimble, D. M. Bunting, A. S. Fawole, M. Basheer, R. V. Dave, J. Sarveswaran, E. Jones, C. J. Kendall, M. P. Tilston, M. Gough, T. Wallace, S. Singh, J. Downing, K. A. Mockford, E. Issa, N. Shah, N. Chauhan, T. R. Wilson, A. Forouzanfar, J. R. Wild, E. Nofal, C. Bunnell, K. Madbak, S. T. Rao, L. Devoto, N. Siddiqi, Z. Khawaja, J. C. Hewes, L. Gould, A. Chambers, D. Urriza Rodriguez, G. Sen, S. Robinson, K. Carney, F. Bartlett, D. M. Rae, T. E. Stevenson, K. Sarvananthan, S. J. Dwerryhouse, S. M. Higgs, O. J. Old, T. J. Hardy, R. Shah, S. T. Hornby, K. Keogh, L. Frank, M. Al-Akash, E. A. Upchurch, R. J. Frame, M. Hughes, C. Jelley, S. Weaver, S. Roy, T. O. Sillo, G. Galanopoulos, T. Cuming, P. Cunha, S. Tayeh, S. Kaptanis, M. Heshaishi, A. Eisawi, M. Abayomi, W. S. Ngu, K. Fleming, D. Singh Bajwa, V. Chitre, K. Aryal, P. Ferris, M. Silva, S. Lammy, S. Mohamed, A. Khawaja, A. Hussain, M. A. Ghazanfar, M. I. Bellini, H. Ebdewi, M. Elshaer, G. Gravante, B. Drake, A. Ogedegbe, D. Mukherjee, C. Arhi, L. Giwa Nusrat Iqbal, N. F. Watson, S. Kumar Aggarwal, P. Orchard, E. Villatoro, P. D. Willson, K. Wa, J. Mok, T. Woodman, J. Deguara, G. Garcea, B. I. Babu, A. R. Dennison, D. Malde, D. Lloyd, S. Satheesan, O. Al-Taan, A. Boddy, J. P. Slavin, R. P. Jones, L. Ballance, S. Gerakopoulos, P. Jambulingam, S. Mansour, N. Sakai, V. Acharya, M. M. Sadat, L. Karim, D. Larkin, K. Amin, A. Khan, J. Law, S. Jamdar, S. R. Smith, K. Sampat, K. M O’shea, M. Manu, F. M. Asprou, N. S. Malik, J. Chang, M. Johnstone, M. Lewis, G. P. Roberts, B. Karavadra, E. Photi, J. Hewes, L. Gould, A. Chambers, D. Rodriguez, D. A. O’Reilly, A. J. Rate, H. Sekhar, L. T. Henderson, B. Z. Starmer, P. O. Coe, S. Tolofari, J. Barrie, G. Bashir, J. Sloane, S. Madanipour, C. Halkias, A. E. Trevatt, D. W. Borowski, J. Hornsby, M. J. Courtney, S. Virupaksha, K. Seymour, S. Robinson, H. Hawkins, S. Bawa, P. V. Gallagher, A. Reid, P. Wood, J. G. Finch, J. Parmar, E. Stirland, J. Gardner-Thorpe, A. Al-Muhktar, M. Peterson, A. Majeed, F. M. Bajwa, J. Martin, A. Choy, A. Tsang, N. Pore, D. R. Andrew, W. Al-Khyatt, C. T. Bhandari, A. Chambers, D. Subramanium, S. K. Toh, N. C. Carter, S. J. Mercer, B. Knight, S. Tate, B. Pearce, D. Wainwright, V. Vijay, S. Alagaratnam, S. Sinha, S. Khan, S. S. El-Hasani, A. A. Hussain, V. Bhattacharya, N. Kansal, T. Fasih, C. Jackson, M. N. Siddiqui, I. A. Chishti, I. J. Fordham, Z. Siddiqui, H. Bausbacher, I. Geogloma, K. Gurung, G. Tsavellas, P. Basynat, A. Kiran Shrestha, S. Basu, A. Chhabra Mohan Harilingam, M. Rabie, M. Akhtar, P. Kumar, S. F. Jafferbhoy, N. Hussain, S. Raza, M. Haque, I. Alam, R. Aseem, S. Patel, M. Asad, M. I. Booth, W. R. Ball, C. P. Wood, A. C. Pinho-Gomes, A. Kausar, M. Rami Obeidallah, J. Varghase, J. Lodhia, D. Bradley, C. Rengifo, D. Lindsay, S. Gopalswamy, I. Finlay, S. Wardle, N. Bullen, S. Y. Iftikhar, A. Awan, J. Ahmed, P. Leeder, G. Fusai, G. Bond-Smith, A. Psica, Y. Puri, D. Hou, F. Noble, K. Szentpali, J. Broadhurst, R. Date, M. R. Hossack, Y. Li Goh, P. Turner, V. Shetty, M. Riera, C. A. Macano, A. Sukha, S. R. Preston, J. R. Hoban, D. J. Puntis, S. V. Williams, R. Krysztopik, J. Kynaston, J. Batt, M. Doe, A. Goscimski, G. H. Jones, S. R. Smith, C. Hall, N. Carty, J. Ahmed, S. Panteleimonitis, R. T. Gunasekera, A. R. Sheel, H. Lennon, C. Hindley, M. Reddy, R. Kenny, N. Elkheir, E. R. McGlone, R. Rajaganeshan, K. Hancorn, A. Hargreaves, R. Prasad, D. A. Longbotham, D. Vijayanand, I. Wijetunga, P. Ziprin, C. R. Nicolay, G. Yeldham, E. Read, J. A. Gossage, R. C. Rolph, H. Ebied, M. Phull, M. A. Khan, M. Popplewell, D. Kyriakidis, A. Hussain, N. Henley, J. R. Packer, L. Derbyshire, J. Porter, S. Appleton, M. Farouk, M. Basra, N. A. Jennings, S. Ali, V. Kanakala, H. Ali, R. Lane, R. Dickson-Lowe, P. Zarsadias, D. Mirza, S. Puig, K. Al Amari, D. Vijayan, R. Sutcliffe, R. Marudanayagam, Z. Hamady, A. R. Prasad, A. Patel, D. Durkin, P. Kaur, L. Bowen, J. P. Byrne, K. L. Pearson, T. G. Delisle, J. Davies, M. A. Tomlinson, M. A. Johnpulle, C. Slawinski, A. Macdonald, J. Nicholson, K. Newton, J. Mbuvi, A. Farooq, B. Sidhartha Mothe, Z. Zafrani, D. Brett, J. Francombe, P. Spreadborough, J. Barnes, M. Cheung, A. Z. Al-Bahrani, G. Preziosi, T. Urbonas, J. Alberts, M. Mallik, K. Patel, A. Segaran, T. Doulias, P. A. Sufi, C. Yao, S. Pollock, A. Manzelli, S. Wajed, M. Kourkulos, R. Pezzuto, M. Wadley, E. Hamilton, S. Jaunoo, R. Padwick, M. Sayegh, R. C. Newton, M. Hebbar, S. F. Farag, J. Spearman, M. F. Hamdan, C. D’Costa, C. Blane, M. Giles, M. B. Peter, N. A. Hirst, T. Hossain, A. Pannu, Y. El-Dhuwaib, T. E. Morrison, G. W. Taylor, R. L. Thompson, K. McCune, P. Loughlin, R. Lawther, C. K. Byrnes, D. J. Simpson, A. Mawhinney, C. Warren, D. McKay, C. McIlmunn, S. Martin, M. MacArtney, T. Diamond, P. Davey, C. Jones, J. M. Clements, R. Digney, W. M. Chan, S. McCain, S. Gull, A. Janeczko, E. Dorrian, A. Harris, S. Dawson, D. Johnston, B. McAree, E. Ghareeb, G. Thomas, M. Connelly, S. McKenzie, K. Cieplucha, G. Spence, W. Campbell, G. Hooks, N. Bradley, A. D. Hill, J. T. Cassidy, M. Boland, P. Burke, D. M. Nally, A. D. Hill, E. Khogali, W. Shabo, E. Iskandar, G. P. McEntee, M. A. O’Neill, C. Peirce, E. M. Lyons, A. W. O’Sullivan, R. Thakkar, P. Carroll, I. Ivanovski, P. Balfe, M. Lee, D. C. Winter, M. E. Kelly, E. Hoti, D. Maguire, P. Karunakaran, J. G. Geoghegan, S. T. Martin, F. McDermott, K. S. Cross, F. Cooke, S. Zeeshan, J. O. Murphy, K. Mealy, H. M. Mohan, K. Nedujchelyn, M. Fahad Ullah, I. Ahmed, F. Giovinazzo, J. Milburn, S. Prince, E. Brooke, J. Buchan, A. M. Khalil, E. M. Vaughan, M. I. Ramage, R. C. Aldridge, S. Gibson, G. A. Nicholson, D. G. Vass, A. J. Grant, D. J. Holroyd, M. A. Jones, C. M. Sutton, P. O’Dwyer, F. Nilsson, B. Weber, T. K. Williamson, K. Lalla, A. Bryant, C. R. Carter, C. R. Forrest, D. I. Hunter, A. H. Nassar, M. N. Orizu, K. Knight, H. Qandeel, S. Suttie, R. Belding, A. McClarey, A. T. Boyd, G. J. Guthrie, P. J. Lim, A. Luhmann, A. J. Watson, C. H. Richards, L. Nicol, M. Madurska, E. Harrison, K. M. Boyce, A. Roebuck, G. Ferguson, P. Pati, M. S. Wilson, F. Dalgaty, L. Fothergill, P. J. Driscoll, K. L. Mozolowski, V. Banwell, S. P. Bennett, P. N. Rogers, B. L. Skelly, C. L. Rutherford, A. K. Mirza, T. Lazim, H. C. Lim, D. Duke, T. Ahmed, W. D. Beasley, M. D. Wilkinson, G. Maharaj, C. Malcolm, T. H. Brown, G. M. Shingler, N. Mowbray, R. Radwan, P. Morcous, S. Wood, A. Kadhim, D. J. Stewart, A. L. Baker, N. Tanner, H. Shenoy, S. Hafiz, J. A. De Marchi, D. Singh-Ranger, E. Hisham, P. Ainley, S. O’Neill, J. Terrace, S. Napetti, B. Hopwood, T. Rhys, J. Downing, S. Kanavati, M. Coats, D. Aleksandrov, C. Kallaway, S. Yahya, B. Weber, A. Templeton, M. Trotter, C. Lo, A. Dhillon, N. Heywood, Y. Aawsaj, A. Hamdan, O. Reece-Bolton, A. McGuigan, Y. Shahin, A. Ali, A. Luther, J. A. Nicholson, I. Rajendran, M. Boal, J. Ritchie

**Affiliations:** Trent Oesophago-Gastric Unit, Nottingham University Hospitals NHS Trust, Nottingham City Hospital, Hucknall Road, Nottingham, NG5 1PB UK

## Abstract

**Background:**

Day-case surgery is associated with significant patient and cost benefits. However, only 43% of cholecystectomy patients are discharged home the same day. One hypothesis is day-case cholecystectomy rates, defined as patients discharged the same day as their operation, may be improved by better assessment of patients using standard preoperative variables.

**Methods:**

Data were extracted from a prospectively collected data set of cholecystectomy patients from 166 UK and Irish hospitals (CholeS). Cholecystectomies performed as elective procedures were divided into main (75%) and validation (25%) data sets. Preoperative predictors were identified, and a risk score of failed day case was devised using multivariate logistic regression. Receiver operating curve analysis was used to validate the score in the validation data set.

**Results:**

Of the 7426 elective cholecystectomies performed, 49% of these were discharged home the same day. Same-day discharge following cholecystectomy was less likely with older patients (OR 0.18, 95% CI 0.15–0.23), higher ASA scores (OR 0.19, 95% CI 0.15–0.23), complicated cholelithiasis (OR 0.38, 95% CI 0.31 to 0.48), male gender (OR 0.66, 95% CI 0.58–0.74), previous acute gallstone-related admissions (OR 0.54, 95% CI 0.48–0.60) and preoperative endoscopic intervention (OR 0.40, 95% CI 0.34–0.47). The CAAD score was developed using these variables. When applied to the validation subgroup, a CAAD score of ≤5 was associated with 80.8% successful day-case cholecystectomy compared with 19.2% associated with a CAAD score >5 *(p *< 0.001).

**Conclusions:**

The CAAD score which utilises data readily available from clinic letters and electronic sources can predict same-day discharges following cholecystectomy.

## Introduction

Laparoscopic cholecystectomy is the treatment of choice for most patients with symptomatic cholelithiasis and one of the most commonly performed general surgical operations [1]. Following laparoscopic cholecystectomy, most patients can be safely discharged home on the day of the surgery with no difference in outcomes when day of surgery (day-case) discharges are compared with patients who stay overnight [2]. Despite this, wide variation exists in the rates of day-case cholecystectomy across hospitals and countries ranging from 40 to 83% [3]. A recent audit in the UK found that only 12% of hospitals met the current target of 75% of cholecystectomy as day case despite a vision that inpatient cholecystectomy should be the exception rather than the default [4]. The reasons for this are likely to be multifactorial, with patient, surgical, anaesthetic and organisational factors influencing this [5, 6].

There have been evolutions and streamlining of both anaesthetic and surgical techniques to help facilitate same-day discharges [4]. Other strategies have focused on improving patient selection with variable results. A reliable, objective, cost-effective and reproducible method to help improve day-case cholecystectomy rates is needed. The aim of this study is to use a validated national UK database to develop and validate a score to predict successful day-case cholecystectomy operations.

## Methods

Data for this study were derived from the CholeS study, a multicentre, prospective population-based cohort study of variation of cholecystectomy [7]. Data were collected from 8913 patients undergoing cholecystectomy in 166 hospitals across the UK and Ireland, during a 2-month period from March to April 2014. The data were found to be 99.2% accurate by independent data validation [7]. Data were collected prospectively by surgical trainees, who formed a network of surgical research collaborative groups across the UK. Emergency cholecystectomy operations that were performed during the emergency admission were excluded. Preoperative variables included age at the time of operation, body mass index (BMI), primary diagnosis/indication for cholecystectomy, and American Society of Anaesthesiologists (ASA) scores of 1, 2 and ≥3. Preoperative imaging was grouped into abdominal ultrasound scan (USS) only, other radiological imaging and endoscopic investigations. USS reported gallbladder wall thickening and common bile duct (CBD) dilatation were also recorded. The definition of a ‘day-case operation’ used here was a discharge occurring the same day as the operation.

The data set was analysed using Stata, StataCorp. 2017. Stata Statistical Software: Release 15, College Station, TX: StataCorp LLC. Continuous variables were found to be skewed, and so were reported as medians and interquartile ranges (iqr), with Mann–Whitney tests used to compare the two groups. Nominal variables were compared between the groups using Fisher’s exact test, where this was calculable, or with Chi-square test where this was not possible, whilst Kendall’s tau was used to compare ordinal variables. The data were then randomly divided 3:1 into main and validation data sets, respectively. Within the main data set, univariable analyses were used to compare predictors of failed day-case cholecystectomy operation and preoperative factors that influence this. Multivariate logistic regression modelling was then used to assess the impact of preoperative variables on outcome and the coefficient multiplied by two and rounded to the nearest integer in order to develop a predictive risk score. The score was applied to the main and validation data sets and receiver operating characteristic (ROC) and area under the curve (AUC) analysis performed to assess validity and accuracy. Missing data were excluded from the analysis, and *p *< 0.05 was considered statistically significant.

## Results

The CholeS data set included 8913 consecutive cases, 7426 (83.3%) of which were performed electively. Day-case cholecystectomy was performed in 3662 (49.3%) of the elective cases. The median length of hospital stay for those who were admitted to hospital was 1 (iqr 1–2) days. The data set was divided into main (*n* = 5569) and validation (*n* = 1857) data sets. Of the 2687 patients that were not discharged on the day of surgery, 1477 (55.1%) were intended to be day case. However, 2748 (95.5%) of the 2882 patients that had a successful same-day discharge were planned to be day case. The median waiting time in days from listing was longer for the non-day-case group compared with those that were discharged on the day of surgery, 74 (iqr 41,125) and 71 (iqr 42,110), respectively, *(p *< 0.047). Demographics from the main data set are presented in Table 1.

**Table 1:** Comparison preoperative patient and clinical factors between day-case and non-day-case cholecystectomy

Patient and preoperative factors	Day case (*n* = 2882)^a^	Non-day case (*n* = 2687)^a^	*p* value
Age category (years): *n* (%)			
<30	512 (17.8)	233 (8.7)	<0.001
30–40	434 (15.1)	285 (10.6)	
41–50	601 (20.9)	474 (17.7)	
51–60	616 (21.4)	564 (21.0)	
61–70	498 (17.3)	580 (21.6)	
71+	220 (7.6)	549 (20.5)	
Gender: *n* (%)			
Male	616 (21.4)	785 (29.4)	<0.001
Female	2268 (78.6)	1900 (70.6)	
ASA: (%)			
ASA 1	1358 (47.5)	797 (29.9)	<0.001
ASA 2	1376 (48.1)	1461(54.8)	
ASA 3+	128 (4.5)	407 (15.3)	
BMI (%)			
<17.9	10 (0.4)	10 (0.5)	0.118
18–25	579 (21.0)	535 (20.8)	
25–30	1007 (36.5)	907 (35.3)	
31–35	685 (24.8)	579 (23.2)	
36–40	470 (17.0)	513 (20.0)	
>41	8 (0.3)	7 (0.3)	
Previous hospital admission: *n* (%)^b^	1060 (36.8)	1394 (51.9)	<0.001
Primary indication for surgery: *n* (%)			
Biliary colic	1932 (67.0)	1361 (50.8)	<0.001
Cholecystitis	579 (20.1)	768 (29.03)	
Pancreatitis	177 (6.1)	252 (9.4)	
CBD stone	130 (5)	239 (8.9)	
Other	64 (2.2)	49 (1.9)	
Preoperative investigations: *n* (%)^c^			
USS only	2033 (70.5)	1467 (54.7)	<0.001
Radiological	614 (21.3)	789 (29.4)	<0.001
Endoscopic	235 (8.2)	424 (15.8)	<0.001
Ultrasound scan findings: *n* (%)			
Thick-walled gallbladder	698 (24.7)	888 (34.0)	<0.001
Dilated CBD	329 (11.6)	498 (19.0)	<0.001
Consultant speciality: *n* (%)^d^			
HPB/UGI	1761 (61.3)	1564 (58.4)	0.030
Other	1113 (38.7)	1113 (41.6)	

*ASA* American Society of Anesthesiologists physical status classification score, *BMI* body mass index, *CBD* common bile duct

^a^Day-case—same-day hospital discharge, Non-day-case—hospital admission and stay >1 day

^b^Previous gallstone-related emergency admission to hospital

^c^Preoperative investigations: radiological—CT and MR cholangiopancreatography (MRCP); endoscopic—endoscopic retrograde cholangiopancreatography (ERCP) and endoscopic ultrasound

^d^Consultant speciality: HPB—hepatopancreaticobiliary surgery; UGI—upper gastrointestinal surgery/oesophago-gastric surgery; other—colorectal, vascular and breast surgery

Day-case cholecystectomy operations were more likely to fail in males, older patients, higher ASA scores, those who had a previous emergency admission with biliary disease, diagnoses other than biliary colic, whether a thick-walled gallbladder was seen on an ultrasound, those requiring more advanced radiological or endoscopic interventions or whether a non-UGI/HPB consultant was performing the cholecystectomy in both uni- (Table 1) and multivariate (Table 2) analyses. However, BMI was comparable between the groups.

**Table 2:** Multivariate logistic regression analysis

Patient and preoperative factors	Coefficient	Odds ratio (95% confidence interval)	*p* value
Age category (years)			
<30	–	–	–
30–40	− 0.367	0.693 (0.559 to 0.859)	0.001
41–50	− 0.550	0.577 (0.474 to 0.702)	<0.001
51–60	− 0.699	0.497 (0.410 to 0.603)	<0.001
61–70	− 0.940	0.391 (0.321 to 0.475)	<0.001
71+	− 1.702	0.182 (0.146 to 0.227)	<0.001
Gender			
Female	–	–	–
Male	− 0.420	0.657 (0.582 to 0.743)	<0.001
ASA			
ASA 1	–	–	–
ASA 2	− 0.593	0.553 (0.493 to 0.620)	<0.001
ASA 3+	− 1.690	0.185 (0.149 to 0.229)	0.000
Previous hospital admission^a^			
No	–	–	–
Yes	− 0.619	0.538 (0.484 to 0.599)	0.599
Primary indication for surgery			
Biliary colic	–	–	–
Cholecystitis	− 0.644	0.525 (0.462 to 0.597)	<0.001
Pancreatitis	− 0.704	0.495 (0.403 to 0.607)	<0.001
CBD stone	− 0.959	0.383 (0.306 to 0.480)	<0.001
Other	− 0.182	0.834 (0.587 to 1.184)	0.309
Preoperative investigations^b^			
USS only			
Radiological	− 0.584	0.558 (0.500 to 0.632)	<0.001
Endoscopic	− 0.924	0.397 (0.336 to 0.472)	<0.001
Ultrasound scan findings			
Normal-walled gallbladder	–	–	–
Thick-walled gallbladder	− 0.454	0.635 (0.564 to 0.714)	<0.001
Normal CBD	–	–	–
Dilated CBD	− 0.580	0.560 (0.482 to 0.652)	<0.001
Consultant speciality^c^			
HPB/UGI	–	–	–
Other	− 0.119	0.888 (0.798 to 0.989)	0.030

*ASA* American Society of Anesthesiologists physical status classification score, *CBD* common bile duct

^a^Previous gallstone-related emergency admission to hospital

^b^Preoperative investigations: radiological—CT and MR cholangiopancreatography (MRCP); endoscopic—endoscopic retrograde cholangiopancreatography (ERCP) and endoscopic ultrasound

^c^Consultant speciality: HPB—hepatopancreaticobiliary surgery; UGI—oesophago-gastric surgery; other—colorectal, vascular and breast surgery

The coefficients of the regression analysis (Table 2) were multiplied by two and rounded to the nearest integer to form the Cholecystectomy As A Day Case (CAAD) score (Table 3). Area under the ROC (AUROC) analysis of the main data set demonstrated sensitivity of 72% and specificity of 53% with a CAAD score of 5 out of 15, 0.663 (95% CI 0.649 to 0.677) (*p *< 0.001). The CAAD score was then added to the validation group, resulting in an AUROC 0.656 (95% CI 0.63 to 0.68) (*p *< 0.001). A CAAD score of ≤5 was associated with 80.8% successful day-case cholecystectomy compared with 19.2% associated with a CAAD score >5 *(p *< 0.001). Furthermore, CAAD score >5 was associated with a 67% reduction in likelihood of successful day-case cholecystectomy.

**Table 3:** Cholecystectomy As A Day Case (CAAD) score

Patient and preoperative factors	Points
Age category (years)	
<30	0
30–60	1
61–70	2
71+	3
Gender	
Female	0
Male	1
ASA	
ASA 1	0
ASA 2	1
ASA 3+	3
Previous admission to hospital^a^	1
Primary indication for surgery	
Biliary colic	0
Cholecystitis	1
Pancreatitis	1
CBD stone	2
Other	0
Preoperative investigations^b^	
USS only	0
Radiological	1
Endoscopic	2
Ultrasound scan findings	
Thick-walled gallbladder	1
Dilated CBD	1

*ASA* American Society of Anesthesiologists physical status classification score, *CBD* common bile duct

^a^Previous gallstone-related emergency admission to hospital

^b^Preoperative investigations: radiological—CT and MR cholangiopancreatography (MRCP); endoscopic—endoscopic retrograde cholangiopancreatography (ERCP) and endoscopic ultrasound

## Discussion

The aim of this study was to develop and validate a score to help predict successful day-case cholecystectomy. The contemporary CholeS data set was utilised and the CAAD score developed using preoperative variables readily available from clinic letters and electronic sources. A CAAD score of ≤5 out of 15 can predict same-day discharges following cholecystectomy in the validation data set. Incorporation of this score into clinical practice could help increase the rates of successful day-case cholecystectomy operation allowing better service planning and bed management.

The present study identified factors that impact on the likelihood of same-day discharge following cholecystectomy, some of which are predictable. Younger age and lower ASA scores are linked with fewer co-morbidities, anaesthetic risks, less intensive perioperative monitoring and as a result higher probability of same-day discharge. There is also evidence to suggest that older age and a higher ASA score are risk factors for cholecystectomy-related complications and conversion to open surgery [8]. However, the present study suggests that patients with high ASA or advanced age, in isolation, can still have successful day-case cholecystectomy; however, if these factors coexist, then same-day discharge is much less likely.

Female gender is also associated with higher rates of successful day-case cholecystectomy; this may be explained by reports suggesting that a greater proportion of male patients have complicated cholelithiasis compared with female patients [9]. Others have also reported that cholecystectomy in men can be technically more challenging and is associated with prolonged operative duration and higher rates of conversion to open and therefore longer postoperative stay [8–10].

Other factors associated with prolonged postoperative stay include: a previous emergency admission with a diagnosis other than biliary colic or thick-walled gallbladder or CBD stones on imaging, again these factors are associated with more complicated operations and increased risk of conversion to open as well as longer postoperative stay [8]. Interestingly, some of these factors are not usually considered to have a significant influence on the likely success of day-case cholecystectomy, particularly if they occur in isolation.

Obesity is believed to be an anaesthetic and perioperative surgical risk factor [11]. Most day-case departments will have varying protocols restricting day-case cholecystectomy in patients with high BMI. However, this current study showed little effect of BMI on likelihood of successful day case. This is consistent with some previous reports suggesting that BMI alone has little influence on the success of day-case cholecystectomy [12].

Surgeon factors also appear to influence day-case success. The lead surgeon’s speciality is linked with the likelihood of same-day discharge with higher rates of successful day-case operations associated with upper gastrointestinal and hepatopancreaticobiliary surgeons; this may reflect volume-related experience.

The CAAD score developed in this study combines the effect of these patient and surgical factors to improve case selection. This simple score is the first prospective validated score to successfully predict day-case cholecystectomy and is derived from easily accessible patient-related data. Such a score could improve service planning, increase day-case surgery rates and increase availability of inpatient beds as well as facilitate significant cost savings. Single-centre studies have integrated protocolled perioperative pathways for day-case gallbladder surgery which result in an increase in successful day-case rate with no detrimental effect in conversion rate or readmission rate [13]. Other scoring systems have investigated factors that result in prolonged postoperative stay following cholecystectomy, but these use intraoperative data which limits their use and applicability in preoperative planning [14, 15].

The present study has limitations. The data taken from the CholeS data set represent a 2-month snapshot of practice [7]. The short intensive data collection allowed surgical teams to contribute meaningful numbers of patients with high levels of accuracy. The primary aim of the CholeS study was to assess the variation in practice of cholecystectomy in the UK and was not designed to develop a risk score to predict day-case operations. As the data were extracted retrospectively, there was no information on factors, which may also influence day-case surgery, such as previous abdominal operations, time surgery was performed, social circumstances or organisational factors.

This score is based on UK data sets, and as such it is not clear as to the applicability to non-UK health systems. However, it is reasonable to assume that the CAAD score may be of interest to other health systems that share similar population groups as well as infrastructure.

The financial benefits of day-case surgery in selected cholecystectomy patients are well established. While it is accepted that multiple factors need to be addressed in order to achieve successful same day discharge, the introduction of the CAAD score could aid this by allowing better selection of patients for day case lists.
