# Correction: The Cholecystectomy As A Day Case (CAAD) Score: A Validated Score of Preoperative Predictors of Successful Day-Case Cholecystectomy Using the CholeS Data Set

**DOI:** 10.1007/s00268-023-06911-y

**Published:** 2023-01-27

**Authors:** A. M. El-Sharkawy, N. Tewari, R. S. Vohra, CholeS Study Group, West Midlands Research Collaborative, R. S. Vohra, S. Pasquali, A. J. Kirkham, P. Marriott, M. Johnstone, P. Spreadborough, D. Alderson, E. A. Griffiths, S. Fenwick, M. Elmasry, Q. Nunes, D. Kennedy, R. Basit Khan, M. A. Khan, C. J. Magee, S. M. Jones, D. Mason, C. P. Parappally, P. Mathur, M. Saunders, S. Jamel, S. Ul Haque, S. Zafar, M. H. Shiwani, N. Samuel, F. Dar, A. Jackson, B. Lovett, S. Dindyal, H. Winter, T. Fletcher, S. Rahman, K. Wheatley, T. Nieto, S. Ayaani, H. Youssef, R. S. Nijjar, H. Watkin, D. Naumann, S. Emeshi, P. B. Sarmah, K. Lee, N. Joji, J. Heath, R. L. Teasdale, C. Weerasinghe, P. J. Needham, H. Welbourn, L. Forster, D. Finch, J. M. Blazeby, W. Robb, A. G. McNair, A. Hrycaiczuk, A. Charalabopoulos, S. Kadirkamanathan, C. B. Tang, N. V. Jayanthi, N. Noor, B. Dobbins, A. J. Cockbain, A. Nilsen-Nunn, J. de Siqueira, M. Pellen, J. B. Cowley, W. M. Ho, V. Miu, T. J. White, K. A. Hodgkins, A. Kinghorn, M. G. Tutton, Y. A. Al-Abed, D. Menzies, A. Ahmad, J. Reed, S. Khan, D. Monk, L. J. Vitone, G. Murtaza, A. Joel, S. Brennan, D. Shier, C. Zhang, T. Yoganathan, S. J. Robinson, I. J. McCallum, M. J. Jones, M. Elsayed, L. Tuck, J. Wayman, K. Carney, S. Aroori, K. B. Hosie, A. Kimble, D. M. Bunting, A. S. Fawole, M. Basheer, R. V. Dave, J. Sarveswaran, E. Jones, C. J. Kendall, M. P. Tilston, M. Gough, T. Wallace, S. Singh, J. Downing, K. A. Mockford, E. Issa, N. Shah, N. Chauhan, T. R. Wilson, A. Forouzanfar, J. R. Wild, E. Nofal, C. Bunnell, K. Madbak, S. T. Rao, L. Devoto, N. Siddiqi, Z. Khawaja, J. C. Hewes, L. Gould, A. Chambers, D. Urriza Rodriguez, G. Sen, S. Robinson, K. Carney, F. Bartlett, D. M. Rae, T. E. Stevenson, K. Sarvananthan, S. J. Dwerryhouse, S. M. Higgs, O. J. Old, T. J. Hardy, R. Shah, S. T. Hornby, K. Keogh, L. Frank, M. Al-Akash, E. A. Upchurch, R. J. Frame, M. Hughes, C. Jelley, S. Weaver, S. Roy, T. O. Sillo, G. Galanopoulos, T. Cuming, P. Cunha, S. Tayeh, S. Kaptanis, M. Heshaishi, A. Eisawi, M. Abayomi, W. S. Ngu, K. Fleming, D. Singh Bajwa, V. Chitre, K. Aryal, P. Ferris, M. Silva, S. Lammy, S. Mohamed, A. Khawaja, A. Hussain, M. A. Ghazanfar, M. I. Bellini, H. Ebdewi, M. Elshaer, G. Gravante, B. Drake, A. Ogedegbe, D. Mukherjee, C. Arhi, L. Giwa Nusrat Iqbal, N. F. Watson, S. Kumar Aggarwal, P. Orchard, E. Villatoro, P. D. Willson, K. Wa, J. Mok, T. Woodman, J. Deguara, G. Garcea, B. I. Babu, A. R. Dennison, D. Malde, D. Lloyd, S. Satheesan, O. Al-Taan, A. Boddy, J. P. Slavin, R. P. Jones, L. Ballance, S. Gerakopoulos, P. Jambulingam, S. Mansour, N. Sakai, V. Acharya, M. M. Sadat, L. Karim, D. Larkin, K. Amin, A. Khan, J. Law, S. Jamdar, S. R. Smith, K. Sampat, K. M O’shea, M. Manu, F. M. Asprou, N. S. Malik, J. Chang, M. Johnstone, M. Lewis, G. P. Roberts, B. Karavadra, E. Photi, J. Hewes, L. Gould, A. Chambers, D. Rodriguez, D. A. O’Reilly, A. J. Rate, H. Sekhar, L. T. Henderson, B. Z. Starmer, P. O. Coe, S. Tolofari, J. Barrie, G. Bashir, J. Sloane, S. Madanipour, C. Halkias, A. E. Trevatt, D. W. Borowski, J. Hornsby, M. J. Courtney, S. Virupaksha, K. Seymour, S. Robinson, H. Hawkins, S. Bawa, P. V. Gallagher, A. Reid, P. Wood, J. G. Finch, J. Parmar, E. Stirland, J. Gardner-Thorpe, A. Al-Muhktar, M. Peterson, A. Majeed, F. M. Bajwa, J. Martin, A. Choy, A. Tsang, N. Pore, D. R. Andrew, W. Al-Khyatt, C. T. Bhandari, A. Chambers, D. Subramanium, S. K. Toh, N. C. Carter, S. J. Mercer, B. Knight, S. Tate, B. Pearce, D. Wainwright, V. Vijay, S. Alagaratnam, S. Sinha, S. Khan, S. S. El-Hasani, A. A. Hussain, V. Bhattacharya, N. Kansal, T. Fasih, C. Jackson, M. N. Siddiqui, I. A. Chishti, I. J. Fordham, Z. Siddiqui, H. Bausbacher, I. Geogloma, K. Gurung, G. Tsavellas, P. Basynat, A. Kiran Shrestha, S. Basu, A. Chhabra Mohan Harilingam, M. Rabie, M. Akhtar, P. Kumar, S. F. Jafferbhoy, N. Hussain, S. Raza, M. Haque, I. Alam, R. Aseem, S. Patel, M. Asad, M. I. Booth, W. R. Ball, C. P. Wood, A. C. Pinho-Gomes, A. Kausar, M. Rami Obeidallah, J. Varghase, J. Lodhia, D. Bradley, C. Rengifo, D. Lindsay, S. Gopalswamy, I. Finlay, S. Wardle, N. Bullen, S. Y. Iftikhar, A. Awan, J. Ahmed, P. Leeder, G. Fusai, G. Bond-Smith, A. Psica, Y. Puri, D. Hou, F. Noble, K. Szentpali, J. Broadhurst, R. Date, M. R. Hossack, Y. Li Goh, P. Turner, V. Shetty, M. Riera, C. A. Macano, A. Sukha, S. R. Preston, J. R. Hoban, D. J. Puntis, S. V. Williams, R. Krysztopik, J. Kynaston, J. Batt, M. Doe, A. Goscimski, G. H. Jones, S. R. Smith, C. Hall, N. Carty, J. Ahmed, S. Panteleimonitis, R. T. Gunasekera, A. R. Sheel, H. Lennon, C. Hindley, M. Reddy, R. Kenny, N. Elkheir, E. R. McGlone, R. Rajaganeshan, K. Hancorn, A. Hargreaves, R. Prasad, D. A. Longbotham, D. Vijayanand, I. Wijetunga, P. Ziprin, C. R. Nicolay, G. Yeldham, E. Read, J. A. Gossage, R. C. Rolph, H. Ebied, M. Phull, M. A. Khan, M. Popplewell, D. Kyriakidis, A. Hussain, N. Henley, J. R. Packer, L. Derbyshire, J. Porter, S. Appleton, M. Farouk, M. Basra, N. A. Jennings, S. Ali, V. Kanakala, H. Ali, R. Lane, R. Dickson-Lowe, P. Zarsadias, D. Mirza, S. Puig, K. Al Amari, D. Vijayan, R. Sutcliffe, R. Marudanayagam, Z. Hamady, A. R. Prasad, A. Patel, D. Durkin, P. Kaur, L. Bowen, J. P. Byrne, K. L. Pearson, T. G. Delisle, J. Davies, M. A. Tomlinson, M. A. Johnpulle, C. Slawinski, A. Macdonald, J. Nicholson, K. Newton, J. Mbuvi, A. Farooq, B. Sidhartha Mothe, Z. Zafrani, D. Brett, J. Francombe, P. Spreadborough, J. Barnes, M. Cheung, A. Z. Al-Bahrani, G. Preziosi, T. Urbonas, J. Alberts, M. Mallik, K. Patel, A. Segaran, T. Doulias, P. A. Sufi, C. Yao, S. Pollock, A. Manzelli, S. Wajed, M. Kourkulos, R. Pezzuto, M. Wadley, E. Hamilton, S. Jaunoo, R. Padwick, M. Sayegh, R. C. Newton, M. Hebbar, S. F. Farag, J. Spearman, M. F. Hamdan, C. D’Costa, C. Blane, M. Giles, M. B. Peter, N. A. Hirst, T. Hossain, A. Pannu, Y. El-Dhuwaib, T. E. Morrison, G. W. Taylor, R. L. Thompson, K. McCune, P. Loughlin, R. Lawther, C. K. Byrnes, D. J. Simpson, A. Mawhinney, C. Warren, D. McKay, C. McIlmunn, S. Martin, M. MacArtney, T. Diamond, P. Davey, C. Jones, J. M. Clements, R. Digney, W. M. Chan, S. McCain, S. Gull, A. Janeczko, E. Dorrian, A. Harris, S. Dawson, D. Johnston, B. McAree, E. Ghareeb, G. Thomas, M. Connelly, S. McKenzie, K. Cieplucha, G. Spence, W. Campbell, G. Hooks, N. Bradley, A. D. Hill, J. T. Cassidy, M. Boland, P. Burke, D. M. Nally, A. D. Hill, E. Khogali, W. Shabo, E. Iskandar, G. P. McEntee, M. A. O’Neill, C. Peirce, E. M. Lyons, A. W. O’Sullivan, R. Thakkar, P. Carroll, I. Ivanovski, P. Balfe, M. Lee, D. C. Winter, M. E. Kelly, E. Hoti, D. Maguire, P. Karunakaran, J. G. Geoghegan, S. T. Martin, F. McDermott, K. S. Cross, F. Cooke, S. Zeeshan, J. O. Murphy, K. Mealy, H. M. Mohan, K. Nedujchelyn, M. Fahad Ullah, I. Ahmed, F. Giovinazzo, J. Milburn, S. Prince, E. Brooke, J. Buchan, A. M. Khalil, E. M. Vaughan, M. I. Ramage, R. C. Aldridge, S. Gibson, G. A. Nicholson, D. G. Vass, A. J. Grant, D. J. Holroyd, M. A. Jones, C. M. Sutton, P. O’Dwyer, F. Nilsson, B. Weber, T. K. Williamson, K. Lalla, A. Bryant, C. R. Carter, C. R. Forrest, D. I. Hunter, A. H. Nassar, M. N. Orizu, K. Knight, H. Qandeel, S. Suttie, R. Belding, A. McClarey, A. T. Boyd, G. J. Guthrie, P. J. Lim, A. Luhmann, A. J. Watson, C. H. Richards, L. Nicol, M. Madurska, E. Harrison, K. M. Boyce, A. Roebuck, G. Ferguson, P. Pati, M. S. Wilson, F. Dalgaty, L. Fothergill, P. J. Driscoll, K. L. Mozolowski, V. Banwell, S. P. Bennett, P. N. Rogers, B. L. Skelly, C. L. Rutherford, A. K. Mirza, T. Lazim, H. C. Lim, D. Duke, T. Ahmed, W. D. Beasley, M. D. Wilkinson, G. Maharaj, C. Malcolm, T. H. Brown, G. M. Shingler, N. Mowbray, R. Radwan, P. Morcous, S. Wood, A. Kadhim, D. J. Stewart, A. L. Baker, N. Tanner, H. Shenoy, S. Hafiz, J. A. De Marchi, D. Singh-Ranger, E. Hisham, P. Ainley, S. O’Neill, J. Terrace, S. Napetti, B. Hopwood, T. Rhys, J. Downing, S. Kanavati, M. Coats, D. Aleksandrov, C. Kallaway, S. Yahya, B. Weber, A. Templeton, M. Trotter, C. Lo, A. Dhillon, N. Heywood, Y. Aawsaj, A. Hamdan, O. Reece-Bolton, A. McGuigan, Y. Shahin, A. Ali, A. Luther, J. A. Nicholson, I. Rajendran, M. Boal, J. Ritchie

**Affiliations:** Trent Oesophago-Gastric Unit, Nottingham University Hospitals NHS Trust, Nottingham City Hospital, Hucknall Road, Nottingham, NG5 1PB UK

**Correction: World J Surg (2019) 43:1928–1934** 10.1007/s00268-019-04981-5

Since the publication of this work, S. Kanavati has changed their name from O. Kanavati. This has now been amended.

